# MosaicIA: an ImageJ/Fiji plugin for spatial pattern and interaction analysis

**DOI:** 10.1186/1471-2105-14-349

**Published:** 2013-12-03

**Authors:** Arun Shivanandan, Aleksandra Radenovic, Ivo F Sbalzarini

**Affiliations:** 1 MOSAIC Group, Center of Systems Biology Dresden (CSBD), Max Planck Institute of Molecular Cell Biology and Genetics, Pfotenhauerstr 108, 01307 Dresden, Germany; 2 Previously: MOSAIC Group, Department of Computer Science, ETH Zurich, 8092 Zurich, Switzerland; 3 Laboratory of Nanoscale Biology, Institute of Bioengineering, School of Engineering, EPFL, 1015 Lausanne, Switzerland

**Keywords:** Spatial pattern analysis, Microscopy, Co-localization analysis, Interaction analysis, PALM, ImageJ, Fiji, Image analysis

## Abstract

**Background:**

Analyzing spatial distributions of objects in images is a fundamental task in many biological studies. The relative arrangement of a set of objects with respect to another set of objects contains information about potential interactions between the two sets of objects. If they do not “feel” each other’s presence, their spatial distributions are expected to be independent of one another. Spatial correlations in their distributions are indicative of interactions and can be modeled by an effective interaction potential acting between the points of the two sets. This can be used to generalize co-localization analysis to spatial interaction analysis. However, no user-friendly software for this type of analysis was available so far.

**Results:**

We present an ImageJ/Fiji plugin that implements the complete workflow of spatial pattern and interaction analysis for spot-like objects. The plugin detects objects in images, infers the interaction potential that is most likely to explain the observed pattern, and provides statistical tests for whether an inferred interaction is significant given the number of objects detected in the images and the size of the space within which they can distribute. We benchmark and demonstrate the present software using examples from confocal and PALM single-molecule microscopy.

**Conclusions:**

The present software greatly simplifies spatial interaction analysis for point patterns, and makes it available to the large user community of ImageJ and Fiji. The presented showcases illustrate the usage of the software.

## Background

We present a software plugin to analyze and quantify spatial patterns of objects in images using the free open-source image-processing platform ImageJ [1] or its distribution Fiji [2]. The spatial arrangement of objects relative to each other is a rich source of phenotypic information. This ranges from spatial patterns of sub-cellular structures or proteins, to the spatial patterns formed by cells in tissues, to spatial patterns of organisms in ecosystems. The mathematical framework of spatial statistics allows quantifying and analyzing such patterns, comparing them with each other, and performing statistical tests on them [3-5]. This for example allows testing whether the distribution of a set of objects is significantly different from random, or whether the objects in one set are distributed independently of the objects in another set. Significant deviations from spatial randomness are indicative of interactions (of some sort) between the objects, as formalized in the framework of spatial interaction analysis [6].

Biology has long relied on co-localization analysis in order to quantify the spatial distribution of one set of objects with respect to another one. This includes pixel-based and object-based co-localization analysis methods [7]. Pixel-based methods typically use a correlation measure between the pixel intensities in different images in order to quantify the degree of overlap or co-localization between the object distributions represented in the images. Object-based methods first detect and delineate the objects of interest in the images and then quantify their degree of co-localization using an overlap measure. While pixel-based measures are easy to compute, they are difficult to interpret. They are also sensitive to blurring and noise in the image. Moreover, in some observations, like those from Photo-Activation Localization Microscopy (PALM) and STochastic Optical Reconstruction Microscopy (STORM) [8-10], it is not obvious what constitutes an image [11], as these methods provide locations and localization uncertainties of individual molecules. Pixel-based methods are hence not directly applicable, unless one first renders a synthetic image from the observed point locations. This, however, leads to a loss of information as nearby molecule detections fuse into one blob in the rendered image. Object-based methods are more intuitive to interpret, as they directly work with locations of objects. However, they depend on prior object detection and segmentation. It is also not clear when two object should be considered “overlapping”. This requires defining a distance threshold, which typically is ≈200 nm for diffraction-limited data [12]. Single-molecule imaging, like PALM and STORM, directly provides point locations, rendering a separate object-detection step unnecessary.

While co-localization analysis captures the amount of overlap between objects, it is not sufficient to describe spatial patterns and interactions [6]. Also, co-localization measures do not account for the fact that accidental overlap occurs even in randomly distributed objects and becomes more frequent as the object density increases. This typically leads to a density bias in the final co-localization score.

### Spatial interaction analysis

**Figure 1:**
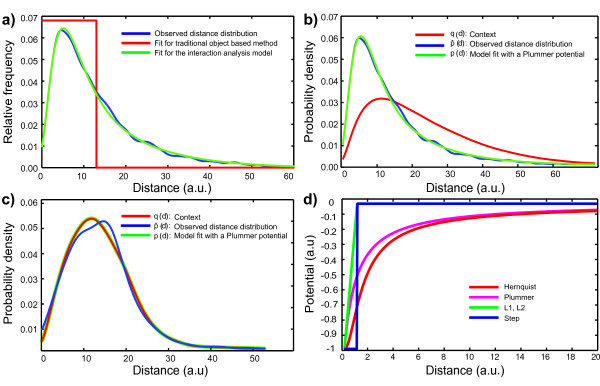
**Interaction analysis with smooth potentials and context correction. (a)** Example comparison of fits to data (blue) using traditional, uncorrected object-based co-localization analysis (red) and using the context-corrected method used in MosaicIA (green). The plots show the nearest-neighbor (NN) distance distribution between two arbitrary point patterns. **(b,c)** Sample fits corresponding to interacting and non-interacting point patterns, respectively. In the case of the non-interacting point pattern, the model fit (green) to the observed NN distribution (blue) coincides with the context (red). In the presence of an interaction, the NN distance distribution (blue) is different from the context (red). The difference is explained by an interaction potential, leading to the model fit (green). **(d)** Shapes of different potentials provided by MosaicIA. Parameters: *σ* =v1, and *t* = 0 for all except step potential, where *σ* = *t* = 1.

Helmuth et al. [6] have generalized co-localization analysis to interaction analysis, which corrects for accidental overlaps, is robust against imaging noise and image-processing errors, does not require defining a distance threshold, and is able to capture patterns also at larger length scales than just immediate overlap. They used a nearest-neighbor interaction model based on spatial Gibbs statistics, which characterizes an observed pattern by an interaction potential. This is the potential that is most likely to produce the observed distribution of nearest-neighbor (NN) distances between objects if the objects interact pair-wise according to this potential. Compared to object-based co-localization analysis, the interaction analysis model uses additional information that is present in the data, namely nearest-neighbor distance distributions also between non-overlapping objects. The model is hence able to better fit an observed distance distribution than a co-localization score would be (see Figure 1a). The method also provides standardized ways to (1) correct for the influence of the distribution of points within one set onto the distance distribution to another set; (2) infer parameters of the interaction potential, such as the strength and length scale of the interaction; (3) perform statistical hypothesis tests for the presence of an interaction.

The notion of “interaction” used here is purely geometric. We say that two sets of objects interact if the spatial distribution of one set is not independent of the distribution of the other set (see Figure 1b). The objects do not interact if the distribution of one set can be explained by a random distribution that does not depend on the other, the reference set (see Figure 1c). These interactions or spatial patterns need not imply causal interactions.

### The Interaction analysis model

Let the two sets of objects be represented by their spatial positions $X={\left\{x_{i}\right\}}_{i=1}^{N}$ and $Y={\left\{y_{j}\right\}}_{j=1}^{M}$. Further let $D={\left\{d_{i}\right\}}_{i=1}^{N}$ be the set of distances from any point in *X* to its nearest neighbor (NN) in the reference set *Y*. The context *q*(*d*) is the probability density function (p.d.f.) of NN distances if the objects in *X* were distributed uniformly at random and independently of the distribution of the objects in *Y*. We call this the *context* because it corrects for how the objects in *Y* are distributed and for the shape of the space within which the objects in *X* can distribute. The p.d.f. of the observed NN distances is then modeled as [6]:

(1) $$p(D|q)=Z^{-N}\overset{N}{\underset{i=1}{\prod }}q(d_{i})\exp\left(-\phi (d_{i})\right)\;,$$

where *Z* is the normalization constant (partition function), and *ϕ*(*d*) the unknown distance-dependent interaction potential. The role of the potential is to “deform” the context in order to explain the distribution of *X* with respect to *Y* as a result of the points in *X* interacting with their nearest neighbors in *Y* through the potential *ϕ* (see Figure 1b,c).

The potential *ϕ*(*d*) can be of any shape, including a step function for which a context-corrected version of traditional threshold-based co-localization measure is recovered [6]. Often, we use a parametric potential of the form *ϕ*(*d*) = *εf*((*d* - *t*) / *σ*), where *ε* is the interaction strength, *σ* the length scale of the interaction, and *t* a threshold closer than which objects are considered overlapping. The function *f* defines the shape of the potential. If the shape *f* is unknown, a piece-wise linear non-parametric function is used. Plots of the different parametric potential shapes provided by the plugin are shown in Figure 1d.

## Implementation

The plugin is written in Java. It uses the open-source Java library Weka [13] for efficiently computing NN distances using *kd*-trees and for kernel density estimation of probability distributions. The plugin further uses CMA-ES (the Covariance Matrix Adaptation Evolution Strategy) [14] for parameter optimization. This optimizer is less prone to get stuck in local optima than the simplex method used in the original publication [6]. The plugin has been tested with both ImageJ and Fiji on Windows, MacOS X, and Linux. It should run on any platform where Java and ImageJ are available. Running a complete analysis using the present plugin takes between a few seconds and a few minutes, depending on the number of objects present in the image.

**Figure 2:**
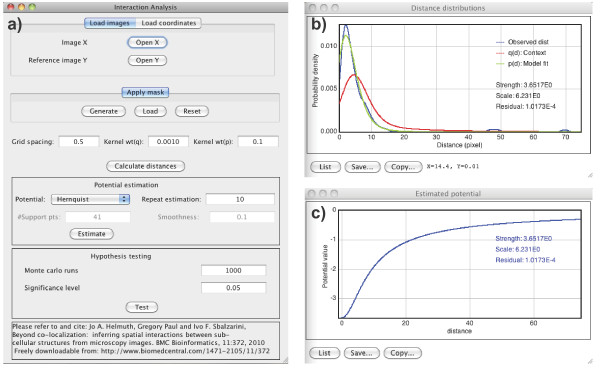
**The graphical user interface of the plugin. (a)** The main mask of MosaicIA where the parameters are entered. **(b,c)** Windows showing example results of an analysis. The measured and fitted NN distance distributions, along with the context, are shown in **(b)**. The interaction potential leading to the displayed fit is shown in **(c)**.

**Figure 3:**
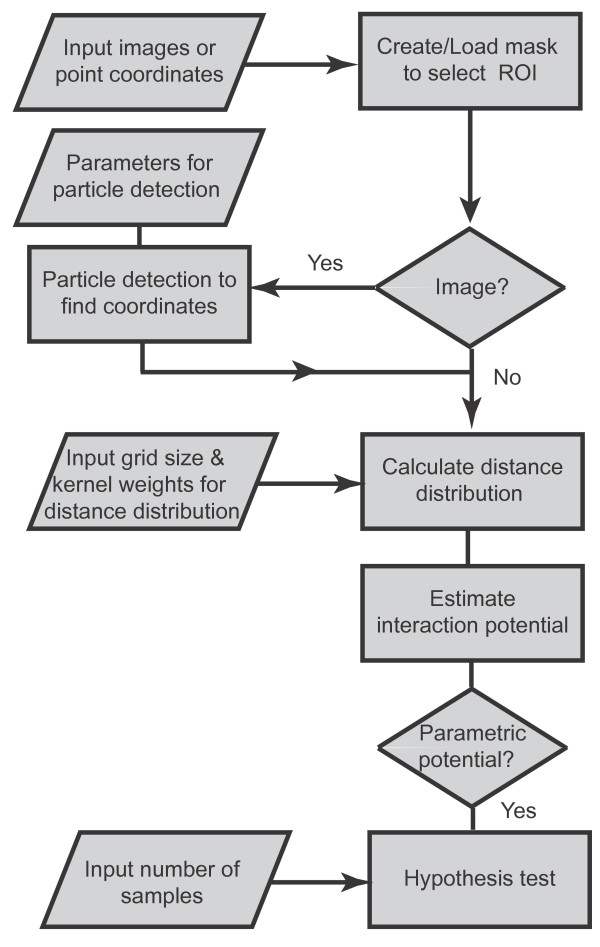
**Workflow of interaction analysis with MosaicIA.** See main text for details and examples.

The user interface of MosaicIA is shown in Figure 2. Its workflow is explained in the flowchart in Figure 3. The plugin reads either images (2D or 3D), or comma-separated text files containing the coordinates of objects. The user can create or load a mask to restrict the analysis to a certain region of interest, if necessary. If the analysis is based on images, bright points in the images are first detected using the feature-point detector by Sbalzarini and Koumoutsakos [15], which is also available in Java as an ImageJ plugin and processes both 2D and 3D images. Then, the NN distance distribution *D* between the two point sets is computed and the context *q*(*d*) estimated using grid sampling [6]. This means that *q*(*d*) is approximated by computing the NN distance from each point on a regular Cartesian lattice to the nearest neighbor in *Y*. The grid resolution has to be set by the user. Finer grids lead to more accurate results, but require more computer time. Smooth representations of the observed (empirical) NN distance distribution $\hat{p}(d)$, and of the sampled context *q*(*d*) are obtained by kernel density estimation. The estimator kernel widths are set by the user, but the software provides an initial guess calculated with Silverman’s rule [16].

The plugin currently works with point objects only, even though the interaction analysis framework generalizes to extended objects too [6]. In order to work with non-point objects in the present plugin, they can be segmented using other software, e.g. the Region Competition plugin for ImageJ [17], and their centers of mass can be read into the present plugin. Representing extended objects by their centers of mass does not significantly change the result of the interaction analysis, as shown in the PALM example below.

The type of the potential function can be selected from a drop-down menu. Both parametric and non-parametric functions are provided. The plugin then estimates the potential or its parameters from the data by minimizing the *ℓ*^2^ difference $∥\hat{p}(d)-p(d){∥}_{2}$ between the observed NN distance distribution $\hat{p}(d)$ and the one predicted from the interaction model *p*(*d*). The results, including the residual fitting error, are then shown along with a plot of $\hat{p}(d),p(d)$, and *q*(*d*), as shown as in Figure 2b,c.

The plugin also provides hypothesis tests to check whether the estimated interaction is statistically significant. The test statistic is computed from *K* Monte Carlo samples of point distributions corresponding to the null hypothesis of “no interaction”. The test statistic from the actually observed distribution is ranked against these *K* random samples. If it ranks higher than ⌈(1 - *α*)*K*⌉-th, the null hypothesis of “no interaction” is rejected on the significance level *α*[6].

### List of parameter inputs to the plugin

The plugin has six parameters that the user can set to control its behavior. These parameters and their typical values are described below.

#### Parameters for object detection

The following parameters control the image-processing part of the plugin. The algorithm used to detect bright objects (blobs) in the images and extract their location is described in Ref. [15]. See this reference for a more in-depth discussion of these parameters and for illustrations of how they affect object detection. 

• **Radius**: Approximate radius of the blobs in the images in units of pixels. This value should be slightly larger than the visible object radius, but smaller than the smallest NN object separation.

• **Cutoff**: The score cut-off for false-positive rejection. The larger the cutoff, the more conservative the algorithm becomes to only select objects that look alike.

• **Percentile**: Determines how bright a blob has to be in order to be considered an object. All local intensity maxima in the given upper percentile of the image intensity histogram are considered candidate objects.

#### Parameters for computing distance distributions

Once the objects have been detected in both images, or their coordinates have been read from files, the following parameters can be used to control interaction inference: 

• **Grid spacing**: The grid spacing controls how finely the context *q*(*d*) is sampled in units of pixels. It should ideally be less than half of shortest possible interaction that can be detected with the available data. For an image without sub-pixel object detection, 0.5 (pixel) is hence sufficient. In cases where finer resolution is needed, the user can try successively smaller values until the context *q*(*d*) does not change any more. Grid sampling the context *q*(*d*) is the most time-consuming part of the analysis. Adjusting the grid size hence significantly influences the computational time.

• **Kernel wt(q)**: This is the weight parameter used by the kernel density estimator to estimate the smooth context p.d.f. *q*(*d*) from the grid samples. Since the number of grid points is usually large, a small kernel weight of 0.001 should be sufficient to produce smooth results. This parameter usually does not need to be changed.

• **Kernel wt(p)**: This is the weight parameter used by the kernel density estimator to estimate the smooth NN distance distribution $\hat{p}(d)$. The value of this parameter is critical, and a rough estimate for it is computed using Silverman’s rule [16]. The resulting value is shown as a suggestion. This parameter should be carefully tuned so that the resulting distribution contains all relevant information from the histogram, without overfitting it. A larger value for this parameter leads to a more fine-grained, less smooth fit.

### List of potentials provided

The plugin provides both parametric and non-parametric potentials that can be used to describe an interaction. The non-parametric potential is more flexible and does not require the user to assume anything about the functional shape of the interaction. However, it requires more computer time to be estimated and does not support statistical tests for the significance of an interaction. Parametric potentials offer an intuitive interpretation of an interaction by its strength and length scale. Frequently, one first estimates a non-parametric potential in order to get an idea of the rough shape of the interaction. Then, one selects the parametric potential most similar to it and repeats the estimation.

#### Parametric potentials

Potentials are parameterized as *ϕ*(*d*) =v*εf*((*d* - *t*) / *σ*) with interaction strength *ε*, length scale *σ*, and a hard core *t*. For the step potential, *σ* = 1. For all other potentials, *t* = 0. The shapes *f*(·) of the various potentials are: 

• Step potential:

(2) $$f^{\text{st}}(r)=\left\{\begin{array}{ll} -1 & \text{if}\;r<0\\ 0 & \text{else}. \end{array}\right)$$

• Hernquist potential:

(3) $$f^{\text{he}}(r)=\left\{\begin{array}{ll} -{\left(r+1\right)}^{-1} & \text{if}\;r>0\\ -(1-r) & \text{else}. \end{array}\right)$$

• Linear potential, type 1:

(4) $$f^{\text{l}1}(r)=\left\{\begin{array}{ll} 0 & \text{if}\;r>1\\ -(1-r) & \text{else}. \end{array}\right)$$

• Linear potential, type 2:

(5) $$f^{\text{l}2}(r)=\left\{\begin{array}{ll} 0 & \text{if}\;r>1\\ -1 & \text{if}\;r<0\\ -(1-r) & \text{else}. \end{array}\right)$$

• Plummer potential:

(6) $$f^{\text{pl}}(r)=\left\{\begin{array}{ll} -{\left(r^{2}+1\right)}^{-0.5} & \text{if}\;r>0\\ -1 & \text{else}. \end{array}\right)$$

Plots of these potentials are shown in Figure 1d. Other potentials can easily be implemented, if needed.

#### Non-parametric potential

The non-parametric potential does not assume any specific shape and can be used to gain an approximate idea of the shape of an unknown interaction. It is defined as a weighted sum of linear kernel functions centered on *P* support points (defined in the #**support pts** field). The more support points are used, the finer the potential is resolved, but the more costly and unstable the estimation becomes. The smoothness of the estimated potential is controlled by the **smoothness** parameter, which penalizes differences between adjacent support points. Larger smoothness parameters lead to smoother potentials, but may miss or average out interesting interactions. Therefore, this parameter should be used with caution.

### Working with coordinates instead of images

It is possible to directly use MosaicIA on localization data. This is useful when working with imaging modalities like PALM and STORM that provide point coordinates rather than images. It is also useful when working with objects that are not blob-like, or for which the object-detection step of the plugin does not work well. These objects can be detected and segmented using any other tool, e.g. the Region Competition [17] or the split-Bregman/Squassh [18] plugins for ImageJ/Fiji, and their coordinates stored in a file. A comma-separated text file of object coordinates can be read into MosaicIA by clicking ***load coordinates*** instead of ***load images***. Each line in the file should contain the coordinates of one object in the format *x*, *y*, (*z*). The spatial boundaries of the point patterns (they must be identical for both *X* and *Y*) are entered in the fields provided. For objects detected in a 400×400 pixel image, the boundaries are (0, 399) in both directions.

### Interpreting the results

The estimated interaction potentials and parameters can be used to quantitatively compare spatial distributions across different samples and conditions (e.g., perturbations). Comparisons based on interaction strengths and length scales, however, should only be done for results obtained with the same potential shape.

The strength of an interaction, *ε*, is equal to zero for independent, i.e. non-interacting, point patterns. However, due to noise and random overlap in the data, the strength may be slightly greater than zero even in the case of no interaction. A hypothesis test is therefore provided in order to check whether an estimated interaction is statistically significant given the amount and quality of the data used to infer it.

The hard core of an interaction, *t*, is akin to the distance threshold in classical object-based co-localization analysis. If two objects are closer to each other than this hard core, they are considered overlapping.

The length scale of an interaction, *σ*, quantifies the units of length in which the potential is scaled. It hence provides information about the length scale of organization between the two point patterns. The unit of length is pixels if the objects are detected from images. If coordinates are read from a file, the unit of length is as defined in that file.

## Results and discussion

We validated and tested the plugin on synthetically generated point distributions in the presence and absence of interactions and confirmed that interactions were correctly detected (results not shown). We show here the application of the plugin to two real-world cases: interactions between viruses and endosomes in HER-911 cells as inferred from fluorescence confocal microscopy images, and clathrin–GPCR interactions as inferred from PALM super-resolution data in HeLa cells.

### Application to virus–endosome interaction from confocal images

We apply the plugin to analyze the interactions between human adenoviruses of serotype 2 (Ad2), stained with ATTO-647, and Rab5-EGFP-stained endosomes in HER-911 cells (image data: Greber lab, University of Zurich). Similar data has also been used in Ref. [6].

**Figure 4:**
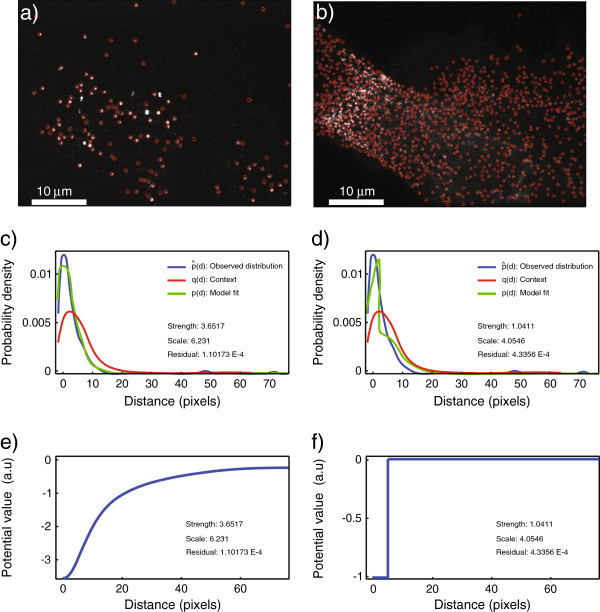
**Results of applying the plugin to virus–endosome data from confocal microscopy. (a)** Image *X* of the red channel showing adenovirus serotype 2 (Ad2) tagged with ATTO-647. **(b)** Image *Y* of the green channel showing Rab5-EGFP, a marker for endosomes. The results from object detection using MosaicIA are shown as overlaid red circles. Only a single 2D image is used here, and no *z*-stack. **(c,d)** Distance distributions obtained after fitting the data with a Plummer and step potential model, respectively. **(e,f)** The corresponding estimated interaction potentials. The Plummer potential leads to a 4-fold lower fitting error than the step potential.

The results are shown in Figure 4. Figure 4a shows the image *X* of the virus particles after object detection in the plugin. The image *Y* of the endosomes after object detection is shown in Figure 4b. Figure 4c shows the observed NN distance distribution (blue curve), the expected distribution if viruses were distributed at random and independently of the endosomes, i.e., the context (red curve), and the best fit (green curve) with the Plummer potential shown in Figure 4e. Figure 4d shows the results when using the step potential shown in Figure 4f, corresponding to a context-corrected object-based co-localization count. The residual fitting error when using the Plummer potential is about 4-fold lower than when using the step potential, even though the latter also corrects for the context. The improvement stems from using continuous distance information. The error when using classical co-localization analysis without correcting for the context would be even larger.

### Application to GPCR–clathrin interaction from PALM data

Super-resolution microscopy techniques such as PALM and STORM do not provide images, but produce point clouds by measuring the coordinates of individual fluorophores and their localization uncertainties. Directly working with these position data provides more information than first rendering an artificial image from them and then working with that image [11].

G-protein-coupled receptors (GPCRs) are important signaling proteins that are transported in clathrin-coated vesicles. The sizes of these vesicles are typically below the resolution limit of classical microscopy, rendering them a good system to be studied with super-resolution techniques like PALM and STORM.

**Figure 5:**
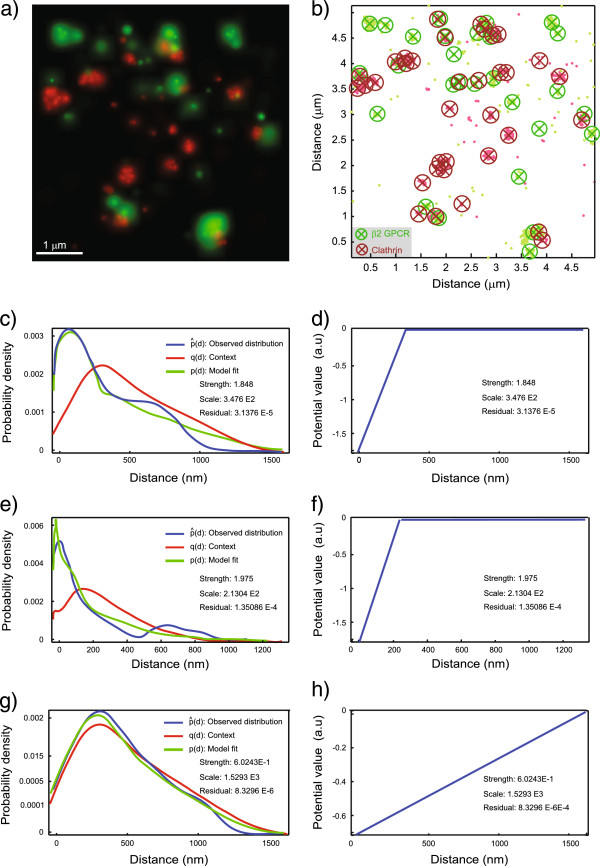
**Results of applying the plugin to clathrin–*β*2-AR data from single-molecule PALM.** MosaicIA applied to PALM super-resolution imaging in fixed HeLa cells: The green channel (*X*) shows the GPCR protein *β*2-AR labelled with PSCFP2. The red channel (*Y*) shows Clathrin Light Chain-PAMCherry1. **(a)** Rendering of the PALM image as a probability map showing only molecules that localized into clusters of a given threshold size. **(b)** These molecules displayed as points without their corresponding localization uncertainty. Clusters of molecules are visualized by circles with × marking the cluster centers. **(c,e,g)** Distance distributions obtained after fitting the model with a linear L1 potential. **(c,d)** Fit and estimated interaction potential when using only cluster centers for the analysis. **(e,f)** Fit and estimated interaction potential when only using all individual molecules. **(g,h)** Randomization control using a randomly shuffled point pattern *X* with the same number of points as in c.

We analyze the prototypical GPCR *β*2-adrenergic receptor (*β*2-AR) and its internalization in clathrin-coated vesicles post stimulation with the agonist isoproterenol in HeLa cells [19]. *β*2-AR is labelled with PSCFP2, and clathrin light chains are labeled with PAMCherry1 [19]. Figure 5a shows an exemplary rendered probability map from dual-color PALM after setting a clustering threshold to remove localized molecules that are not within a cluster of at least the threshold size [19]. The estimated locations of these individual fluorescent molecules are shown as dots in Figure 5b, without the localization uncertainty distributions. Circles mark clusters of fluorophores with the cluster centers given by the crosses.

We can either analyze the interactions between the individual molecules in one color channel with those in the other channel, or we may exploit the biological knowledge that the actual interaction acts at the organelle level rather than the molecular level. For the latter, we hence analyze the interactions between the centers of the detected clusters across the two color channels. For the sake of simplicity, we do not explicitly model localization uncertainty, registration errors, and limited detection efficiency.

The results corresponding to the parametric potential that provided the best fit (in this case an L1 linear potential) are shown in Figure 5. Figures 5c,d show the results of the analysis based on cluster centers, Figures 5e,f those based on individual molecules. In both cases, the residual fitting error is 5-fold lower than when using a step potential. Figures 5g,h show controls obtained by randomly scrambling the cluster-center locations. We see that: (1) the plugin correctly infers an interaction (estimated *ε*=1.85) in the data with cluster centers, but detects no interaction in the randomized control (*ε*=0.6; the null hypothesis of no interaction has rank 512 of 1000 and cannot be rejected). (2) The results when applying the analysis to individual molecules or to cluster centers are similar, with the former estimating *ε*=2.0, indicating that the analysis is robust against clustering effects and correctly identifies the length scale of the interaction. A randomization control based on individual molecules yields *ε*=0.3 (data not shown), and the null hypothesis of no interaction cannot be rejected (rank 202 of 1000). This indicates that analyzing interactions between organelles by considering their cluster centers, rather than individual molecules, may be sufficient to distinguish an interacting pattern from a non-interacting one. Analysis with other potential shapes provided similar results.

## Conclusions

We presented MosaicIA, an ImageJ/Fiji plugin for spatial point pattern and interaction analysis. The plugin takes as an input two 2D or 3D images showing the spatial distributions of two sets of bright, spot-like objects, or it reads the coordinates of two sets of objects from files. It then uses a nearest-neighbor Gibbs interaction model from spatial statistics in order to infer the pair-wise interaction potential that is most likely to create the observed distribution of objects. Compared to classical pixel-based or object-based co-localization analysis, this makes better use of the information present in the image and hence provides superior statistical detection power [6]. The analysis also accounts for the context created by the shape of the space within which the objects are distributed and by the object distribution within the reference set. Estimating interaction models is more robust against imaging noise and image-processing errors than classical co-localization analysis [20]. Statistical tests are provided by the plugin in order to check the significance of an interaction. The estimated interaction parameters provide a quantitative way of comparing spatial patterns across different samples, conditions, and perturbations.

The plugin has been tested on both synthetic and real-world data. We demonstrated its application to virus trafficking data obtained with confocal fluorescence microscopy and to PALM super-resolution data of the interaction between clathrin and *β*2-AR. In the latter case, we compared the results from applying the analysis to individual points with the results obtained from cluster centers. In all tested cases, the best interaction potential explained the data 4 to 5-fold better than a step potential, i.e., than context-corrected object-based co-localization analysis. Without context correction, the fits would be even worse.

Despite the fact that we have here only demonstrated the plugin for fluorescence microscopy and PALM data, it can be applied to distributions of any type of objects (organelles, cells, organisms), as long as their positions can be extracted from images or read from files. For example, in the case of cells in a tissue, it can be applied with the help of segmentation methods that can provide the spatial location that best fits a cell [21]. Comparing the model fits obtained with different parametric potentials can also be used to test and compare hypothetical interaction mechanisms directly on the data.

Future work includes extending the plugin to also handle extended objects that are not blob-like, and to explicitly account for localization uncertainties and registration (aberration) errors. Other useful extensions could be testing for spatial randomness within a single set of objects from the intra-set NN distance histogram, and automatic estimation of algorithm parameters from the data.

## Availability and requirements

The present plugin is available as part of the MOSAICsuite for ImageJ and Fiji.

**Project name:** MosaicIA **Project home page:**http://mosaic.mpi-cbg.de/?q=downloads/imageJ**Fiji update site:**http://mosaic.mpi-cbg.de/Downloads/update/Fiji/MosaicToolsuite**Operating system(s):** Linux, MacOS X, Windows **Programming language:** Java **Other requirements:** ImageJ or Fiji **License:** GNU LGPL v3. Please cite this paper as well as Ref. [6] in any publications that use this software. **Any restrictions to use by non-academics:** licenseneeded

## Competing interests

The authors declare that they have no competing interests.

## Authors’ contributions

Designed the method and the software: IFS and AS, implemented the software: AS, performed the data analysis: AS and AR, wrote the paper: AS, IFS, and AR. All authors read and approved the final manuscript.
